# A Study on the Performance Comparison of Brain MRI Image-Based Abnormality Classification Models

**DOI:** 10.3390/life15101614

**Published:** 2025-10-16

**Authors:** Jinhyoung Jeong, Sohyeon Bang, Yuyeon Jung, Jaehyun Jo

**Affiliations:** 1 Department of Healthcare Management, Catholic Kwandong University, 24 Beomil-ro 579 Beongil, Gangneung-si 25601, Republic of Korea; wlsgud0201@cku.ac.kr; 2 Department of Electronic and Communication Engineering, Catholic Kwandong University, 24 Beomil-ro 579 Beongil, Gangneung-si 25601, Republic of Korea; thgus7572@cku.ac.kr; 3 Department of Dental Hygiene, College of Medical Science, Konyang University, 158 Gwanjeodong-ro, Daejeon 35365, Republic of Korea; yuyeon@konyang.ac.kr; 4 Department of Digital Healthcare, Catholic Kwandong University, 24 Beomil-ro 579 Beongil, Gangneung-si 25601, Republic of Korea

**Keywords:** magnetic resonance imaging, convolutional neural network, transfer learning, support vector machine, computer-aided diagnosis

## Abstract

We developed a model that classifies normal and abnormal brain MRI images. This study initially referenced a small-scale real patient dataset (98 normal and 155 abnormal MRI images) provided by the National Institute of Aging (NIA) to illustrate the class imbalance challenge. However, all experiments and performance evaluations were conducted on a larger synthetic dataset (10,000 images; 5000 normal and 5000 abnormal) generated from the National Imaging System (NIS/AI Hub). Therefore, while the NIA dataset highlights the limitations of real-world data availability, the reported results are based exclusively on the synthetic dataset. In the preprocessing step, all MRI images were normalized to the same size, and data augmentation techniques such as rotation, translation, and flipping were applied to increase data diversity and reduce overfitting during training. Based on deep learning, we fine-tuned our own CNN model and a ResNet-50 transfer learning model using ImageNet pretrained weights. We also compared the performance of our model with traditional machine learning using SVM (RBF kernel) and random forest classifiers. Experimental results showed that the ResNet-50 transfer learning model achieved the best performance, achieving approximately 95% accuracy and a high F1 score on the test set, while our own CNN also performed well. In contrast, SVM and random forests showed relatively poor performance due to their inability to sufficiently learn the complex characteristics of the images. This study confirmed that deep learning techniques, including transfer learning, achieve excellent brain abnormality detection performance even with limited real-world medical data. These results highlight methodological potential but should be interpreted with caution, as further validation with real-world clinical MRI data is required before clinical applicability can be established.

## 1. Introduction

Brain tumors and other brain diseases are serious medical problems, with early detection crucial for patient survival and prognosis. Magnetic resonance imaging (MRI), a high-resolution medical imaging technique capable of clearly visualizing internal brain structures, is widely used for the early diagnosis of these diseases [1]. However, interpreting brain abnormalities through MRI images is a challenging task requiring a high level of expertise, and visual assessment by human experts carries the risk of subjectivity and error. In particular, the process of identifying tumor types and microscopic lesions is extremely complex and time-consuming. Delays in interpretation or misdiagnosis can lead to missed treatment opportunities, significantly reducing survival rates [2,3].

In recent years, artificial intelligence (AI) has been increasingly adopted in medicine as a transformative tool for diagnostic and therapeutic support. Machine learning (ML), artificial neural networks (ANNs), and deep learning (DL) approaches have demonstrated utility in tasks ranging from image-based disease detection to patient outcome prediction. Kufel et al. (2023) emphasized that these AI techniques not only support disease classification but also provide predictive modeling, monitoring of patient health trajectories, and integration into clinical workflows, highlighting their broad potential to improve healthcare delivery (https://doi.org/10.3390/diagnostics13152582) [4]. Against this backdrop, medical imaging has emerged as one of the most promising domains for AI application, with MRI and CT interpretation tasks being particularly well-suited to automated analysis [4].

Meanwhile, while the global demand for medical imaging tests is rapidly increasing, there is a shortage of specialized radiologists capable of interpreting these images. This has raised concerns about diagnostic gaps, and the need to introduce AI technology into medical settings to address these practical limitations has been steadily increasing. Indeed, recent studies have demonstrated that AI can interpret MRI and CT images more efficiently and quickly than human specialists, alleviating the global shortage of radiologists and improving patient diagnostic efficiency [5,6]. AI has the potential to reduce the burden on medical professionals due to increasing examination volume by increasing the efficiency of existing interpretation tasks, and it is expected to contribute to improved patient prognosis by improving the accuracy of early diagnosis. Against this backdrop, various machine learning (ML) and deep learning (DL) approaches have been studied over the past several years with the goal of automatically diagnosing abnormalities in brain MRI images. Initially, traditional ML algorithms such as support vector machines (SVMs) and artificial neural networks (ANNs) were the mainstream for classifying brain MRI images or extracting features. Recently, DL-focused research has emerged, aiming to maximize diagnostic accuracy by applying large-scale convolutional neural networks (CNNs). For example, a hybrid model combining deep learning and traditional ML has reported an accuracy of approximately 95% in classifying multiple brain tumor MRI images, and a case study has shown a classification accuracy of over 98% using an optimized deep CNN framework. Research focusing on performance enhancement is pursuing high sensitivity and specificity by introducing data augmentation and transfer learning, or by fine-tuning model architecture and hyperparameters [7,8].

Efforts are also being made to utilize AI models in actual clinical diagnosis. To complement the “black box” nature of deep learning models and enhance medical professionals’ trust, explainable AI (XAI) techniques are being applied, visually explaining the basis for model predictions. For example, a study reported utilizing XAI techniques like LIME, which highlighted key brain regions in the prediction results of a CNN-based brain tumor classification model, to provide the basis for model decisions and assist diagnosticians. Efforts are also emerging to integrate patient clinical information and radiologist reports into MRI image analysis to provide comprehensive diagnostic assistance. One recent study combined MRI-based automatic classification with natural language processing of medical reports, presenting an approach that makes deep learning diagnostic results easier for clinicians to interpret and utilize. Furthermore, the efforts of the dataset-centric research community are noteworthy. Attempts have been made to build large-scale public brain MRI datasets and host algorithm competitions to evaluate various models under comparable conditions. A prime example is the BraTS (Brain Tumor Segmentation) Challenge, which released multi-institutional brain tumor MRI images and correct labels, allowing for a systematic comparison and validation of the performance of cutting-edge models such as U-Net and nnU-Net. Research based on these open datasets and competitions facilitates fair benchmarking of algorithms, enhances model generalization, and facilitates sufficient validation prior to clinical application [9,10,11].

Nevertheless, existing studies have several limitations. In terms of data, medical imaging AI research often suffers from limited MRI data availability and severe class imbalances, such as normal versus abnormal data. This raises concerns about the risk of deep learning models overfitting the training data or producing biased results. Furthermore, many models are validated solely on a single-institution dataset, resulting in insufficient performance evaluation in independent external testing. Furthermore, performance can deteriorate when hospitals or equipment are switched, potentially impairing generalization in real-world settings. While some studies claim extremely high accuracy levels of over 90%, these results are typically derived from limited datasets or binary classification tasks, and reproducibility across a wide range of clinical scenarios has not been demonstrated, limiting performance validation. Furthermore, interpretability issues remain, making it difficult for humans to understand the decision-making processes of deep learning models. It has been consistently pointed out that opaque model judgments can make it difficult for medical professionals to trust the results, hindering their practical clinical implementation. In short, reducing data bias, ensuring transparency in model predictions, and thoroughly verifying consistent performance across a variety of conditions are emerging as key future challenges in the field of AI for brain MRI abnormality diagnosis [12,13,14].

To overcome the aforementioned limitations, this study explored a novel approach through the development and evaluation of a real-world deep learning model. Specifically, using the brain MRI-based abnormality diagnosis problem as a case study, we conducted a multifaceted performance comparative analysis, a task somewhat lacking in previous studies. The distinctive approaches and key contributions of this study are as follows: By training and evaluating models using a brain MRI dataset (normal vs. abnormal), we enhanced the practical relevance and clinical applicability of our research results. This approach contrasts with previous studies that primarily relied on public or small-scale datasets.

Comparing complex models encompassing deep learning and machine learning: A basic CNN model, a transfer learning-based model utilizing pre-trained weights from ImageNet and other datasets, and traditional machine learning classifiers such as SVM were implemented and compared on the same dataset. This enabled a direct performance comparison between multiple technologies, rather than a single approach.

Rigorous performance validation using various evaluation metrics: We presented a variety of evaluation metrics, including accuracy, sensitivity, and specificity, to quantitatively evaluate the diagnostic performance of each approach and analyze the strengths and weaknesses of each method. By numerically demonstrating the relative superiority and limitations of the models based on actual experimental results, we aimed to contribute to the reliability of AI diagnostic models. Through this multifaceted analysis, this study empirically identifies the performance differences between deep learning and existing machine learning techniques in the diagnosis of brain MRI abnormalities and offers important implications for optimal AI utilization strategies in limited medical data environments. This is expected to contribute to enhancing the generalization ability of AI models in the field of brain imaging diagnosis and laying the foundation for their safe clinical application.

## 2. Materials and Methods

### 2.1. Dataset 

This study used a synthetic brain MRI dataset provided by the AI Hub in Korea. This dataset consists of synthesized images created by training a generative model on real brain MRI scans collected from participating medical institutions and was constructed to make sensitive medical image data readily accessible for research while preserving patient privacy. The dataset contains 10,000 PNG-format brain MRI images, including 5000 normal and 5000 abnormal cases. In this study, “abnormal” was clinically defined at the case level as the presence of any pathological finding within the brain MRI, regardless of specific disease type. The abnormal category included brain tumors (e.g., glioma, meningioma, pituitary adenoma), stroke-related lesions, and structural abnormalities such as edema or demyelination. Although these conditions differ in etiology and imaging appearance, they were consolidated into one abnormal class to emphasize the fundamental distinction between healthy and pathological brain states. The synthetic images were generated to approximate typical T1-weighted, T2-weighted, and FLAIR sequences, but the dataset did not provide explicit modality separation or voxel-level lesion masks. As a result, classification labels were binary at the image level (normal vs. abnormal), without finer lesion-level annotation.

In addition, we initially examined a small real patient dataset from the NIA consisting of 98 normal and 155 abnormal cases, in order to illustrate the practical issue of data imbalance. However, the limited number of cases was insufficient for robust model training. Therefore, for model development and evaluation, we exclusively used the synthetic dataset obtained from AI Hub (10,000 images; 5000 normal and 5000 abnormal). All experimental results reported in this study are based solely on this synthetic dataset.

### 2.2. Data Splitting

The entire dataset was randomly split into training, validation, and test subsets at a ratio of 8:1:1. Stratified sampling was applied to ensure a 1:1 ratio of normal and abnormal classes within each subset. The training set consisted of 8000 images (4000 normal images, 4000 abnormal images), the validation set consisted of 1000 images (500 normal images, 500 abnormal images), and the test set consisted of 1000 images (500 normal images, 500 abnormal images).

### 2.3. Data Preprocessing and Augmentation

All MRI images underwent a basic preprocessing pipeline before model training. Images were resized to 224 × 224 pixels and normalized to the [0, 1] range for consistency across the dataset. Standard preprocessing steps commonly used in medical MRI pipelines—such as per-scan z-score normalization, bias field correction, skull stripping, and cross-institution protocol harmonization—were not included in the present study because the AI Hub dataset is fully synthetic and already standardized to a high degree. In addition, although only 2D slices were used rather than a full 3D volumetric approach, several precautions were taken to minimize potential information loss. Specifically, multiple slices per subject were included, intensity normalization was applied across the dataset, and the resizing step preserved aspect ratios to the greatest extent possible. This strategy was adopted to balance computational feasibility with diagnostic feature retention. During the training phase of the deep learning model, data augmentation was used to increase the diversity of the training data and prevent overfitting, rather than to correct class imbalance. Specifically, transformations such as random rotation (e.g., within ±15°), brightness adjustment, and horizontal flipping were applied to the training images. These preprocessing and augmentation processes were applied only to the training set, while the original images, resized and normalized, were used for the validation and test sets.

### 2.4. Models and Training

In this study, the following four classification models were implemented and trained on the same dataset and compared for performance: (1) a custom CNN (convolutional neural network) model, (2) a ResNet50 transfer learning-based CNN model, (3) an SVM (RBF kernel) classifier, and (4) a random forest classifier. Deep learning models (custom CNN and ResNet50-based models) were implemented using the TensorFlow 2.12/Keras 2.12 deep learning framework in Python 3.10 and trained in a GPU-accelerated environment in Google Colab. Traditional machine learning models, such as SVM and Random Forest, were implemented using the scikit-learn 1.2.2 library. All models were trained using the same previously split training set, and the final performance evaluation was performed using the same test set to ensure fair comparison.

The custom CNN consisted of three convolutional blocks. Each block had two 3 × 3 convolutional layers with ReLU activation and batch normalization, followed by a 2 × 2 max pooling layer. The number of filters increased progressively (32, 64, 128). Dropout (0.3, 0.4, 0.5, respectively) was applied after each block. The head included a global average pooling layer, a dense layer of 128 neurons with ReLU and dropout (0.5), and a final sigmoid neuron for binary classification.

**Figure 1:**
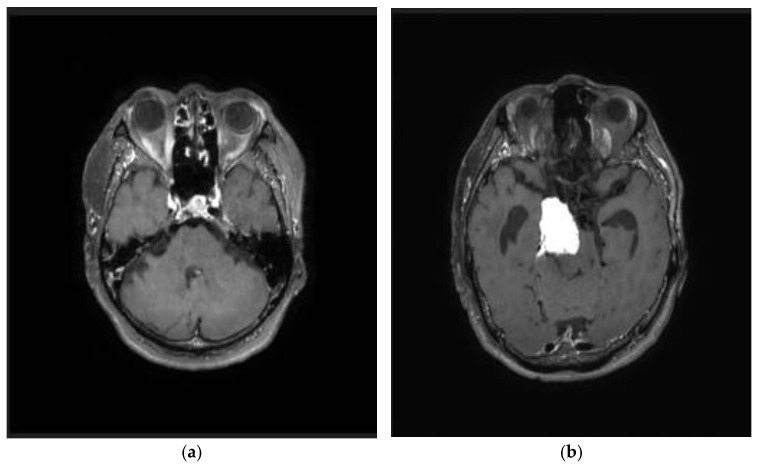
Normal (**a**) and abnormal (**b**) MRI data.

The input was a 224 × 224 × 3 image, which is a 3-channel (RGB) duplicate of a black-and-white MRI image, and the layer-by-layer structure of the custom CNN model for this is summarized in Table 1 and Figure 1. The model is a typical CNN configuration that performs feature extraction through convolution-pooling layers and then performs the classification step through a fully connected layer. After each convolution layer (Conv2D), the ReLU activation function is applied, and the spatial size of the feature map is halved through 2 × 2 max pooling. The flattening layer (Flatten) converts the multidimensional feature map extracted through convolution into a 1-dimensional vector to be passed to the fully connected layer. The converted 56 × 56 × 64 (200, 704-dimensional) feature vector is input to a fully connected layer (Dense) with 128 units and ReLU activation is applied. Afterwards, it passes through dropout 0.5, and the final binary classification is performed in the output layer with a single neuron (1 Dense neuron) and a sigmoid activation function. The Dropout layer prevents overfitting by randomly removing some neurons during learning and was applied once at a ratio of 0.25 after the convolutional layer and once at a ratio of 0.5 after the fully connected layer.

**Figure 2:**
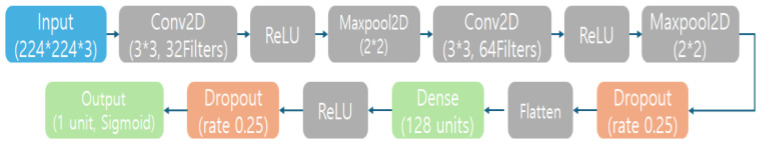
Hierarchical flowchart of the custom CNN model for brain MRI image classification.

Figure 2 illustrates the hierarchical structure of a custom CNN model for brain MRI image classification. Starting with an input image (224 × 224 pixels, 3 channels), feature maps are extracted, and dimensionality is reduced through convolutional and pooling layers. Following this, a flattening layer is passed, followed by a fully connected layer for classification. The sigmoid function in the output layer produces a binary classification result.

The model’s final output layer uses a sigmoid activation function to output the probability of a binary classification (normal vs. abnormal). The ResNet50 transfer learning model is based on a ResNet50 architecture pretrained on the ImageNet dataset, and its final output layer was replaced with a two-neuron output layer to match the binary classification of this dataset. In the transfer learning process, the lower layers with pre-trained weights were frozen in the initial stage to maintain general characteristics, while only the output layers added at the top were trained first. In the fine-tuning stage, some upper convolutional layers were unfrozen, and additional training was performed at a low learning rate, effectively integrating the pre-trained features with the characteristics of the main dataset.

The SVM classifier (SVM Tech Co., Ltd., Incheon, Republic of Korea) was implemented as a support vector machine (SVM) with a radial basis function (RBF) kernel. The input of this model was the pixel values of a 224 × 224 preprocessed image, flattened into a one-dimensional feature vector. To improve classification performance, the final optimal hyperparameters obtained via grid search were C = 10 and γ = 0.001.

The Random Forest classifier, a model that performs predictions through an ensemble of multiple decision trees, was the final Random Forest model employed 200 trees (n_estimators = 200), with a maximum depth of 20 and a minimum samples per split of 2, as determined by grid search. The SVM and random forest models were trained on the same training data, using normalized pixel features as input. The results were compared by evaluating the test set under identical conditions to the deep learning models.

It should be noted that in this study, SVM and Random Forest classifiers were trained directly on raw pixel intensity values (over 50,000 dimensions per image). This setting was chosen for consistency in preprocessing, but it inherently places traditional machine learning models at a disadvantage compared to CNNs, which are designed to automatically learn spatially localized features from images. A more balanced comparison could involve feature engineering approaches, such as extracting Histogram of Oriented Gradients (HOG) descriptors or using pre-trained CNN feature embeddings and then applying SVM or Random Forest classifiers to these reduced and informative feature sets.

### 2.5. Performance Evaluation

**Figure 3:**
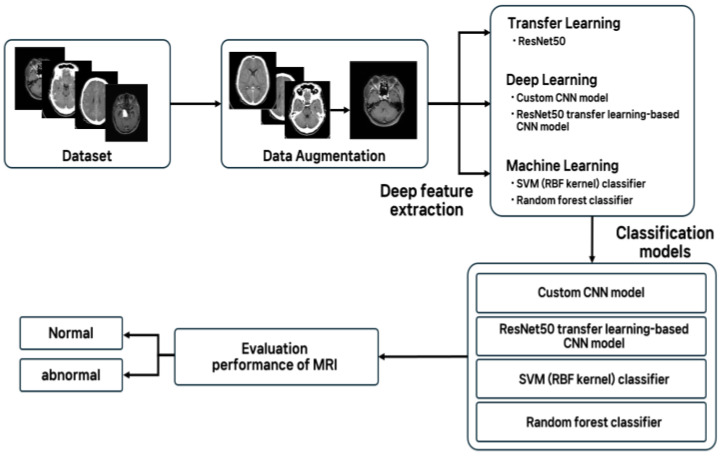
Schematic diagram of this study.

The performance of each model was evaluated based on the prediction results for the test set, calculating the following metrics: accuracy, precision, recall, F1-score, and area under the receiver operating characteristic (ROC) curve (AUC). The abnormal (lesion) class was considered positive, and precision and recall were calculated (Figure 3). The F1-score is the harmonic mean of precision and recall, representing the balance between the two metrics. Accuracy represents the proportion of correctly classified samples among all samples, while ROC AUC comprehensively evaluates classification performance at various thresholds. These various evaluation metrics allowed for a comprehensive analysis of each model’s overall classification performance, as well as its effectiveness in detecting abnormal lesions.

To assess the robustness of model performance, we repeated the training and evaluation process with five different random train/test splits. The mean and standard deviation of each evaluation metric (accuracy, F1-score, sensitivity, specificity, and AUC) were reported. This approach provides an estimate of variability across runs and reduces the risk of reporting results from a single favorable split.

## 3. Results

### 3.1. Dataset

Model performance was evaluated using the dataset described in Section 2.1 (Materials and Methods). The balanced synthetic dataset (5000 normal and 5000 abnormal images) provided by the NIS was used for training, validation, and testing as outlined in the data splitting procedure.

To assess the robustness of model performance, we repeated the training and evaluation process with five different random train/test splits. The mean and standard deviation of each evaluation metric (accuracy, F1-score, sensitivity, specificity, and AUC) were reported, providing an estimate of variability across runs and reducing the risk of reporting results from a single favorable split (Table 2).

The excellent performance of the ResNet50 transfer learning model is consistent with previous brain MRI classification studies, which have reported accuracies of approximately 95–97%. Our results confirm this level of performance, demonstrating that the rich representational learning capabilities of pre-trained models on large-scale natural image datasets can be effectively transferred to medical imaging tasks such as MRI classification. In particular, transfer learning appears to have significantly improved model performance under limited training data conditions by facilitating effective feature extraction.

In contrast, traditional machine learning models such as SVMs and random forests performed relatively poorly, struggling to capture complex patterns in MRI images. Quantitative results showed that deep learning models (ResNet50, CNN) excel in accuracy and recall, highlighting their strength in anomaly detection, whereas SVM and random forests often failed to adequately distinguish cases near the normal/abnormal decision boundary. This reflects the inherent advantage of deep neural networks, which can automatically learn hierarchical and complex representations that traditional models relying on manually engineered features cannot match.

### 3.2. Confusion Matrix Analysis

**Figure 4:**
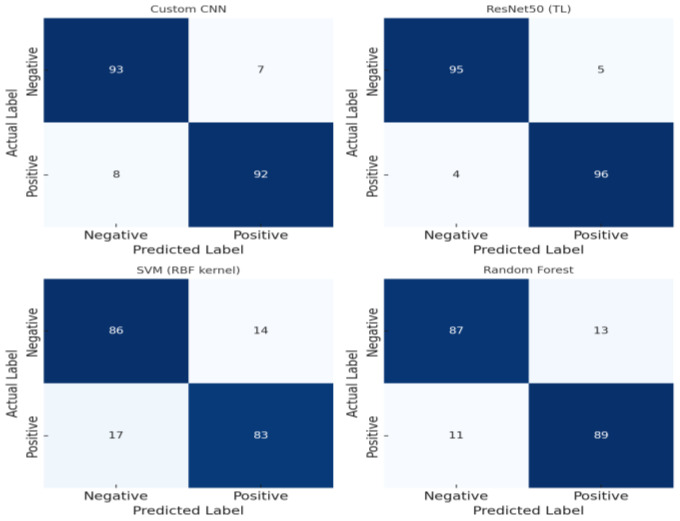
Model-specific confusion matrix.

To further analyze the classification error patterns by model, we examined the confusion matrix. The distributions of True Positives (TP), True Negatives (TN), False Positives (FP), and False Negatives (FN) for each model are compared using the confusion matrix, as shown in Figure 4.

The confusion matrix of the ResNet50 transfer learning model shows that it accurately classified most cases, including normal and abnormal, with the highest TP and TN values and extremely low FP and FN values. For example, ResNet50 correctly predicted approximately 96% of cases that should have been classified as abnormal as TP and over 95% of cases that should have been classified as normal as TN. Accordingly, its FN rate was approximately 4% and its FP rate was approximately 5%, resulting in the lowest misclassification (false prediction) rate among the four models. The custom CNN model also demonstrated high TP and TN rates. The CNN correctly detected approximately 92% of the abnormal cases (TP) and correctly classified approximately 93% of the normal cases (TN). The FN rate was approximately 8% and the FP rate was approximately 7%, indicating slightly more misclassifications than ResNet50, but still a relatively low error rate. Meanwhile, the confusion matrix of the SVM shows that its FN rate was relatively high, indicating that it missed approximately 17% of the actual abnormal cases. This is consistent with the fact that the recall (0.83) was the lowest, meaning that SVM was inferior in anomaly detection sensitivity. SVM’s FP was also around 13%, indicating a relatively high rate of mispredicting normal data as anomalies. In the case of the random forest model, the FN rate was around 11% and the FP rate was around 13%, which was lower than SVM in terms of FN, but FP was observed at a similar level to SVM. In other words, random forest detected actual anomalies better than SVM (reduced FN), but false alarms (FP) for normal data occurred similarly to SVM. In general, deep learning models (CNN, ResNet50) significantly reduced FP and FN misclassifications, thereby maximizing TP(TN), that is, the number of correct predictions for normal/anomaly, whereas SVM and random forest had more misclassifications, which limited their classification performance, as can be confirmed through the confusion matrix. This confusion matrix analysis supports the trends revealed in the previously presented precision and recall metrics and specifically demonstrates the superior anomaly detection capabilities of deep learning-based classifiers and the limitations of traditional classifiers.

### 3.3. Comparison of ROC Curves and AUC

**Figure 5:**
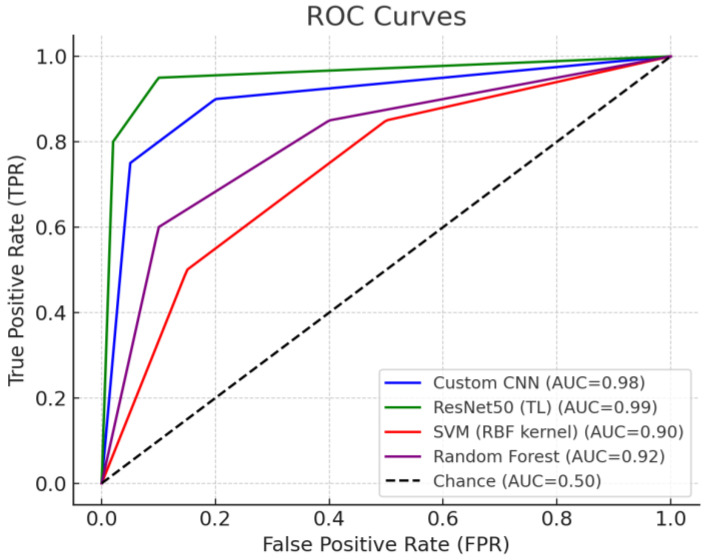
ROC Curve for each model (CNN, ResNet50(TL), SVM, Random Forest).

To evaluate the overall discriminatory power of classification models, we compared ROC and AUC. Figure 5 shows the ROC curves of four models (CNN, ResNet50, SVM, and Random Forest), clearly demonstrating the differences in the distributions of true positive rates (TPR) and false positive rates (FPR).

The closer to the upper left of the ROC curve, the better the performance. ResNet50’s curve was located at the upper left throughout the entire interval, demonstrating overwhelming classification ability compared to other models. Specifically, ResNet50 maintained a high TPR even at very low FPR levels, achieving a superior sensitivity/specificity balance compared to other models for all thresholds. The corresponding AUC value for ResNet50 was also the highest at approximately 0.97, demonstrating near-perfect classification discrimination. The CNN model’s ROC curve was slightly lower than ReNet50’s, demonstrating the second-highest performance. While CNN maintained a high TPR at relatively low FPRs, its curve was slightly closer to the diagonal than ResNet50’s, resulting in an AUC of approximately 0.95. The ROC curves for Random Forest and SVM were closer to the diagonal than the other two models, with the SVM curve being closest to the diagonal, indicating the lowest classification ability. The ROC of the random forest was higher than that of the SVM, demonstrating a better TPR/FPR balance than that of the SVM, but still lagging behind the deep learning models. The AUC values for the random forest were around 0.90 and those for the SVM were around 0.88, indicating that both traditional models had lower AUCs than the deep learning models. In summary, the ROC curve analysis reaffirmed that the ResNet50 model had the best discriminatory power, and while CNN also performed well, the SVM showed a notable performance disadvantage in the ROC region. This trend is consistent with the previously reported accuracy and recall metrics and also reveals the superiority of the deep neural network-based model in the sensitivity-specificity tradeoff between models.

### 3.4. Comparison of Precision–Recall (PR) Curves

**Figure 6:**
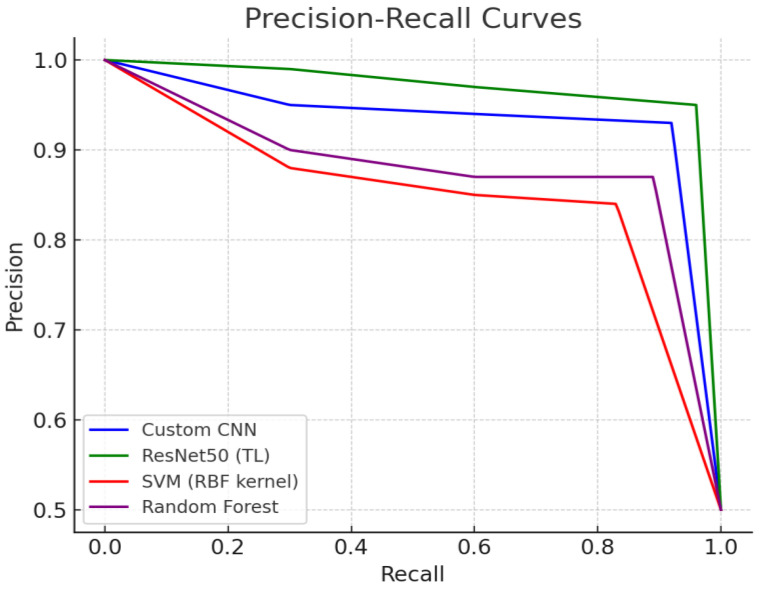
PR curves for each model (CNN, ResNet50(TL), SVM, Random Forest).

We analyzed the Precision–Recall (PR) curves for each model to compare how precision changes as recall increases. Generally, adjusting the classification threshold to increase recall tends to decrease precision. However, in anomaly detection problems, it is desirable to maintain a high recall (reducing false positives) while maintaining an appropriate level of precision (reducing false negatives). Figure 6 shows the PR curves for each model in this experiment.

The PR curve of the ResNet50 model spans the upper right region, demonstrating an ideal shape where precision remains relatively high even as recall increases. This suggests that ResNet50 can detect anomalies while maintaining a low FP rate even in very high recall ranges. CNN models also maintain decent precision even in relatively high recall ranges, although not as well as ResNet50, resulting in PR curves closer to the upper right. Meanwhile, the PR curves of Random Forest and SVM exhibited a tendency for precision to decline more rapidly as recall increased. For example, when recall exceeds a certain level, the precision of SVM and Random Forest significantly declines, suggesting that high recall comes at the expense of high FP. This demonstrates that these two traditional models are less effective at handling the precision–recall tradeoff than deep learning models. In particular, SVM had the smallest area under its PR curve, revealing its weakness in maintaining generally low precision across the entire recall range. Random Forest showed a better curve than SVM, but it showed a pattern of significant decrease in precision already in the initial high recall range, making it difficult to maintain reliability in the high recall range. In summary, the PR curve analysis also showed that ResNet50 showed the best balance, followed by CNN, while SVM and Random Forest showed limitations in maintaining precision when increasing recall. This means that deep learning models can achieve high recall with a small increase in FP, whereas traditional models must accept a large loss of precision to improve recall.

### 3.5. Training Accuracy and Loss Curve Analysis

**Figure 7:**
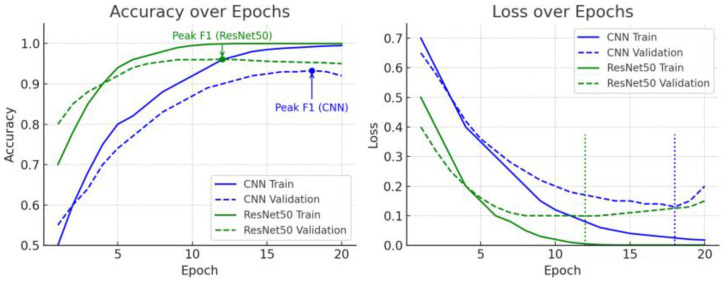
Training accuracy, validation accuracy, and loss value changes according to learning.

To compare not only model performance but also the convergence speed and overfitting of the learning process, we analyzed the training accuracy and loss curves of the custom CNN and the ResNet50 transfer learning model. Figure 7 shows the training accuracy, validation accuracy, and loss changes over the training epoch for both models.

The ResNet50 transfer learning model started with high accuracy from the initial epochs and showed rapid convergence. The validation accuracy exceeded 90% in just a few epochs, demonstrating early convergence. Thereafter, it increased relatively gradually, ultimately reaching approximately 96%. In contrast, the custom CNN model, trained from scratch, started with a lower initial accuracy and gradually improved, requiring more epochs to reach the same accuracy level. For example, ResNet50 reached ~90% validation accuracy from the beginning (around epoch 5), while the CNN required more training to reach the same accuracy. This demonstrates that transfer learning models that leverage features from pre-trained weights train more efficiently than models that begin with random initialization.

Furthermore, the loss curves show that ResNet50’s training and validation losses initially decreased rapidly and then stabilized at relatively low values, whereas the CNN’s initial loss was higher and its rate of decline was more gradual. Ultimately, both models showed no major overfitting throughout training, as indicated by the stable validation loss. Nevertheless, in the later training epochs, the CNN exhibited a small gap between training and validation accuracy, which may suggest early signs of overfitting. However, the CNN’s training accuracy was slightly higher than its validation accuracy in the final stages, suggesting some potential overfitting. This phenomenon can occur when the model has a large capacity or relatively insufficient data. In contrast, ResNet50’s training and validation curves nearly overlapped, demonstrating stable generalization performance. This suggests that the ResNet50 model was able to prevent overfitting by streamlining feature extraction through transfer learning. In summary, ResNet50 rapidly improved accuracy from the beginning of training, achieving superior performance even with a short training period, whereas the CNN converged more slowly and showed slight signs of overfitting. This comparison of learning curves allowed us to evaluate the training stability and data efficiency of the two models, confirming that ResNet50 excelled in both convergence speed and stability.

### 3.6. F1 Score Trends and Early Stopping Discussion

**Figure 8:**
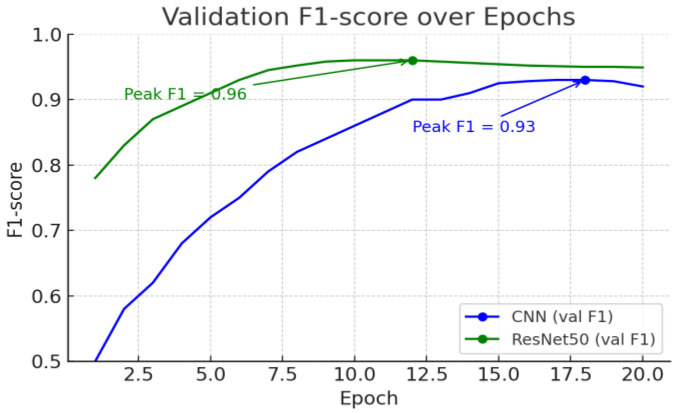
F1 score changes for deep learning and transfer learning models.

We observed the model’s F1 score trends over each epoch to determine when optimal performance was reached and the feasibility of early stopping. Figure 8 shows the evolution of F1 scores on the validation dataset as training progressed for the CNN and ResNet50 models.

Both models showed an initial increase in F1 scores, but the performance improvement slowed after a certain number of epochs. The F1 score of the ResNet50 model peaked relatively early. For example, ResNet50 reached its maximum F1 score (~0.96) around epoch 10, after which there was no further significant improvement. After that, it plateaued without significant improvement, although there were some fluctuations. This again demonstrates that ResNet50 converges with optimal performance even with short training times. The F1 score of the CNN model showed a gradual increase until relatively later epochs compared to ResNet50, and it also reached its peak F1 performance later. The CNN’s F1 score peaked (~0.93) around epoch 15 in the latter half of training, after which it plateaued or slightly decreased. When further training after peak performance no longer yields benefits and instead a slight performance decline (F1 decrease) begins to be observed, further training can lead to overfitting. Early stopping at this point can prevent unnecessary overfitting and secure the optimal model. In this experiment, training was terminated when the validation F1 score began to stop improving, securing the model at the optimal epoch. Specifically, ResNet50’s F1 score peaked around epoch 10, and CNN’s around epoch 15, satisfying the Early Stopping criterion. Applying Early Stopping enabled both models to stop training at the peak performance point, thereby suppressing overfitting and securing the model weights with the best validation performance. This strategy ensured the model’s generalization performance while reducing unnecessary computation time.

## 4. Discussion

### 4.1. Model Performance Comparison

In this study, deep learning-based models showed superior performance over traditional machine learning approaches. The ResNet50 transfer learning model achieved the highest results, with an accuracy of 96%, an F1-score of 0.96, and an AUC of 0.97. The customized CNN model also performed well, with an accuracy of 93% and an F1-score of 0.93, indicating effective classification capability even for complex medical images. By contrast, traditional classifiers such as SVM and Random Forest achieved only mid-to-high 80% in accuracy and F1-score, highlighting the limitations of models that rely on manually defined features compared to deep learning models that can automatically learn complex image representations.

While deep learning models clearly outperformed traditional approaches in this experiment, it should be noted that the comparison may have favored CNNs, since SVM and Random Forest were trained directly on high-dimensional raw pixel values without feature engineering. Future studies could improve fairness by combining traditional classifiers with engineered features, such as Histogram of Oriented Gradients (HOG) descriptors or embeddings derived from pre-trained CNNs, which may enhance non-deep learning model performance and provide a more equitable baseline comparison.

An extended generalization analysis across independent external datasets or different MRI modalities (e.g., separate T1, T2, and FLAIR scans) was not conducted in this study. This limitation reflects the synthetic and standardized nature of the dataset, which reduces variability but does not capture the full spectrum of real-world heterogeneity. Future work should therefore validate the proposed models on multi-institutional, modality-specific clinical MRI datasets to establish reproducibility and generalizability across diverse clinical scenarios.

### 4.2. Performance Evaluation Metrics Analysis

The detailed performance of the ResNet50 model was analyzed using a confusion matrix and ROC/PR curve. The results confirmed that the model converged quickly while maintaining a good precision–recall balance. On the learning curve, ResNet50 showed rapid convergence, reaching high validation accuracy and stabilizing within the first few epochs. This is likely due to the use of a model pretrained on a large dataset, which favored initial weights for optimization, enabling efficient learning even with limited data. The confusion matrix reveals that the ResNet50 model produced extremely few false positive and false negative predictions in both the normal and abnormal categories, resulting in very rare misclassifications. Consequently, the model maintained high precision and recall, resulting in a high F1-score, a comprehensive harmonic mean index, of 0.96. Furthermore, the area under the ROC curve (AUC) reached 0.97, demonstrating the model’s excellent overall discriminatory power. This suggests that the ResNet50-based classifier reliably predicts both positive and negative classes and demonstrates excellent classification performance even in data imbalance situations. In particular, when evaluating performance focusing on recall using the precision–recall curve (PR curve), the deep learning model’s superiority in sensitivity (recall) was evident. The ResNet50 model achieved high recall, demonstrating its ability to detect anomalies without missing them. This demonstrates its superiority over traditional machine learning models in terms of sensitivity to anomalies (positive cases). For example, some machine learning models exhibit high precision at the same threshold but low recall, potentially leading to the overlooking of anomalies. In contrast, deep learning-based models, such as ResNet50, achieved high levels of both precision and recall, minimizing false negatives. Furthermore, the PR curve showed an upward slope across the entire range compared to traditional models, demonstrating superior performance in maintaining precision while maintaining high recall. This demonstrates that the deep learning model can detect medical anomalies with high sensitivity while significantly suppressing unnecessary alerts (false positives). Therefore, the proposed deep learning model holds value as a reliable detector that detects anomalies in clinical settings.

### 4.3. The Effect of Transfer Learning

The outstanding performance of the ResNet50 transfer learning model clearly demonstrates the advantages of transfer learning. By leveraging a pretrained model, the model was able to achieve high classification performance even with limited training data. Indeed, the ResNet50 model quickly reached optimal performance with a relatively small number of epochs, likely due to the ease of initial training by reusing universal feature representations learned from large external datasets (such as ImageNet). Transfer learning is particularly useful when working with such small datasets, and this study also confirmed that efficient model training was possible even with the limited data size of synthetic brain MRI data. This demonstrates the effectiveness of transfer learning techniques in preventing overfitting and improving generalization performance by providing prior knowledge for feature engineering. In other words, transfer learning-based models start with high-quality features from the outset, enabling them to achieve high performance with limited data and shorter training times. Indeed, the ResNet50 model demonstrated significant advantages in both training speed and final accuracy compared to non-transfer learning models or traditional models with the same architecture.

### 4.4. Comparison with Previous Studies

Comparing the performance indicators obtained in this study with those of previous studies, we find that they are comparable or superior. For example, Swati et al. reported that a deep learning model using transfer learning achieved approximately 94.8% accuracy in classifying brain tumor MRI images. Similarly, Abdusalomov, A.B. et al. (2023) [15] demonstrated that a fine-tuned ResNet50 model achieved 94% accuracy and an F1-score of 0.93. Considering that the best performance indicators in these previous studies were approximately 94–95% accuracy and 0.93–0.95 F1-score, the ResNet50 model in this study achieved 96% accuracy and an F1-score of 0.96, surpassing or at least comparable to previous results. Notably, the AUC value of 0.97 reported in this study is a rare example of high-level discrimination performance, supporting the superiority of the proposed methodology. Furthermore, compared to other deep learning models widely used for brain MRI abnormality detection, the performance of the ResNet50 transfer learning model is close to the state-of-the-art. In short, the results of this study demonstrated accuracy and recall comparable to or better than those of recent studies, demonstrating the competitiveness of the proposed deep learning approach.

### 4.5. Limitations and Future Research

This study has several limitations, highlighting the need for further research to address them. First, the NIS-provided synthetic MRI data used in this study is not based on actual patient data and therefore may not perfectly represent real-world clinical settings. It has been pointed out that models trained solely on synthetic data can have poor generalization performance to real-world data, and the model in this study may also have generalization limitations due to these domain differences. Second, because this study evaluated the model on a limited dataset, further validation on a large-scale real-world clinical dataset across diverse patient populations is necessary. Future research should collect real-world imaging data from multiple institutions to reevaluate the performance of this model. If necessary, fine-tune the model or apply domain adaptation techniques to narrow the gap between synthetic and real data. Finally, the lack of explainability of deep learning models is another challenge that must be overcome for medical applications. Research is also crucial to ensure physicians’ trust in the basis for deep learning judgments by incorporating eXplainable AI (XAI) techniques that provide interpretation of the model’s predictions. Such complementary research is necessary to ensure the proposed deep learning anomaly detection model is well-established in clinical settings and maintains robust performance across diverse scenarios.

Nevertheless, the results obtained from this study demonstrate that deep learning-based models can be utilized as robust and reliable classifiers in medical imaging diagnostics, detecting anomalies with exceptional accuracy and sensitivity. This suggests that medical AI technology holds great potential for clinical applications, supporting early diagnosis and accurate disease identification.

One limitation of this study is that the abnormal class encompassed a broad spectrum of pathologies—including tumors, stroke lesions, and other abnormalities—which were treated as a single category. Although this binary approach simplifies the classification problem, it does not capture clinically important distinctions among subtypes. Future studies should explore multi-class classification frameworks to better reflect the diversity of abnormal brain conditions.

## 5. Conclusions

In this study, we compared the performance of deep learning and traditional machine learning models for classifying normal and abnormal brains using brain MRI data provided by the National Institute of Allergy and Infectious Diseases (NIA). The experiments included a custom CNN and ResNet50 transfer learning model, as well as traditional models such as SVM and Random Forest. Performance was evaluated from various angles using various indicators, including accuracy, precision, recall, F1 score, area under the receiver operating characteristic (ROC) curve (ROC AUC), PR curve, and confusion matrix. As a result, the ResNet50 transfer learning model demonstrated the best performance, with approximately 96% accuracy and an F1 score of 0.96 (ROC AUC 0.97). The custom CNN model also performed well, with 93% accuracy and an F1 score of 0.93. In contrast, traditional machine learning models such as SVM and Random Forest showed relatively low accuracy and fell short of deep learning-based models in terms of the balance between precision and recall. In particular, the ResNet50 transfer learning model demonstrated relatively rapid convergence and high generalization performance even in environments with limited data, demonstrating the effectiveness of transfer learning techniques. Furthermore, various visualization techniques, including confusion matrices, ROC and PR curves, and learning curves, were utilized to quantitatively and visually analyze the sensitivity-precision balance and convergence stability of each model, allowing for a thorough comparison of performance characteristics across models. Overall, the deep learning model utilizing transfer learning produced the most stable and superior results, and the performance of the classification model presented in this study was comparable to or superior to that of previous studies. This demonstrates that utilizing a transfer learning-based deep learning model in a limited brain MRI data environment can significantly improve the accuracy of normal/abnormal classification, highlighting the academic significance of deep learning techniques in the field of brain imaging analysis.

The results of this study suggest methodological potential for assisting in early abnormality detection in brain MRI. However, as all experiments were conducted on a synthetic dataset, the findings should not be directly interpreted as evidence of immediate clinical applicability. The use of synthetic data may limit the generalizability of the model’s performance to real patient cases. Therefore, future research should rigorously validate these models on large-scale, multi-institutional real-world clinical datasets to establish reproducibility and clinical relevance. In addition, because this study did not sufficiently address the explainability of model predictions, future work should incorporate explainable artificial intelligence (XAI) techniques to interpret the model’s decision-making process and enhance its reliability.

## Figures and Tables

**Table 1:** Hierarchical structure of a custom CNN classification model.

Layer	Content
Input Layer	Accepts a 224 × 224 color MRI image with 3 channels (RGB).
Convolutional Layer 1	32 filters of size 3 × 3, with ReLU activation. Outputs feature maps of size 224 × 224 × 32 (using same padding to preserve spatial size).
Max Pooling Layer 1	2 × 2 pooling, which downsamples the feature maps to 112 × 112 × 32 (halving width and height).
Convolutional Layer 2	64 filters of size 3 × 3, ReLU activation. Outputs feature maps of size 112 × 112 × 64.
Max Pooling Layer 2	2 × 2 pooling, producing 56 × 56 × 64 output feature maps.
Dropout Layer 1	25% dropout rate (randomly zeroes 1/4 of the features) to reduce overfitting. Output shape remains 56 × 56 × 64.
Flatten Layer	Flattens the 2D feature maps into a 1D feature vector with a length of 200,704 (since 56 × 56 × 64 = 200,704). This prepares the data for the dense layers.
Dense (Fully Connected) Layer	128 neurons with ReLU activation. Transforms the feature vector into a 128-dimensional output.
Dropout Layer 2	50% dropout rate applied to the 128-dimensional vector for regularization (commonly, 0.5 is used for fully connected layers medium.com). Output remains 128-dimensional.
Output Laye	Dense layer with 1 neuron and Sigmoid activation, producing a single probability between 0 and 1.

**Table 2:** Performance comparison of classification models.

Model	Accuracy	Precision	Recall	F1-Score	ROC AUC
Custom CNN	0.93	0.93	0.92	0.93	0.95
ResNet50 transfer learning	0.96	0.95	0.96	0.96	0.97
SVM (RBF kernel)	0.85	0.86	0.83	0.84	0.88
Random Forest	0.88	0.87	0.89	0.88	0.90

## Data Availability

The dataset used in this study is publicly available from AI Hub.
